# Supplementary figures and images for: Retrospective in silico mutation profiling of SARS-CoV-2 structural proteins circulating in Uganda by July 2021: Towards refinement of COVID-19 disease vaccines, diagnostics, and therapeutics
Source: PLoS One. 2022 Dec 22;17(12):e0279428. doi: 10.1371/journal.pone.0279428 (PMC9778641; doi:10.1371/journal.pone.0279428)

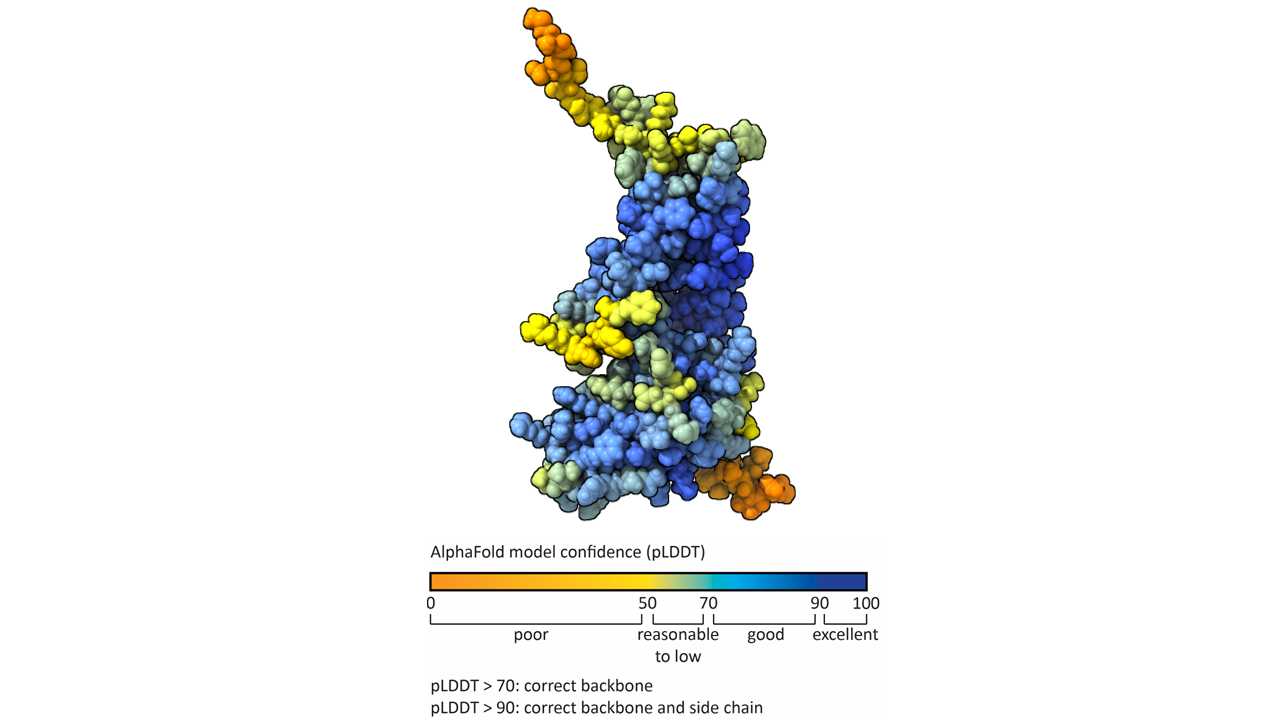

Supplement: S1 Fig — (TIF) [file pone.0279428.s001.tif]

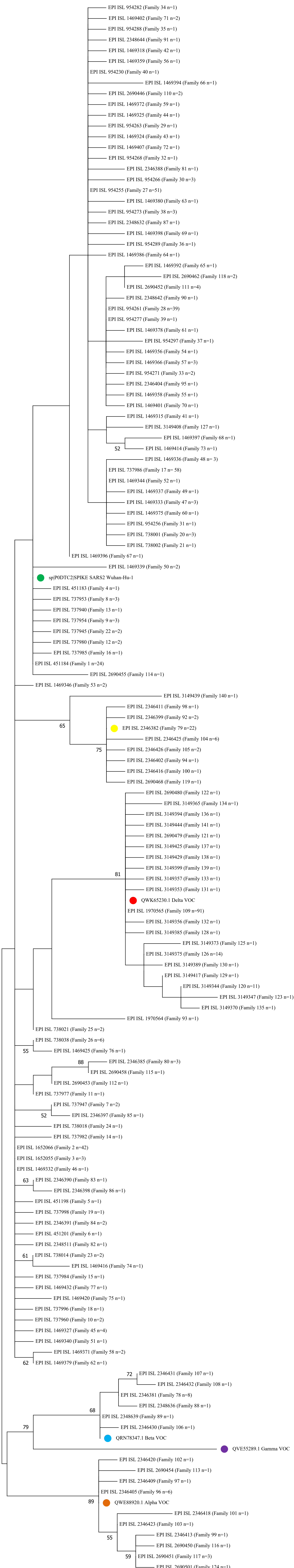

0.0020

Supplement: S2 Fig — Ugandan spike protein sequences clustered with Wuhan-Hu-1 wt (green dot) and three VOCs namely Alpha (orange-brown dot), Beta (blue dot), and Delta (red dot) but not Gamma (magenta dot). Numbers on the branches are bootstrap values. Crowding of the tree was avoided by showing only those bootstrap values >50. There were sequences, which neither clustered with Wuhan-Hu-1 reference strain nor the VOCs (n = 131). Some of these “un-clustered” sequences formed a separate cluster around family 79 (yellow dot). Thus, from largest to smallest cluster we had Wuhan cluster (n = 256), Delta VOC cluster (n = 133), Alpha VOC cluster (n = 17), and Beta VOC cluster (n = 13). (PDF) [file pone.0279428.s002.pdf]
