# Supplementary material for: Retrospective in silico mutation profiling of SARS-CoV-2 structural proteins circulating in Uganda by July 2021: Towards refinement of COVID-19 disease vaccines, diagnostics, and therapeutics
Source: PLoS One. 2022 Dec 22;17(12):e0279428. doi: 10.1371/journal.pone.0279428 (PMC9778641; doi:10.1371/journal.pone.0279428)
Supplement: Attachment — Submitted filename: Reviwers comments.docx [file pone.0279428.s007.docx]

**Reviewers report**

The manuscript, describes of Retrospective in silico mutation profiling of SARS-CoV-2 2 structural proteins circulating in Uganda towards refinement of COVID-19 disease vaccines, 4 diagnostics, and therapeutics

The introduction is very comprehensive and well written. The authors were consistent and applied proof reading skills throughout the document. The methodology section answers the research very well and well detailed. The result were fully described and well detailed.

I find it very fair to congratulate the authors, it was very interesting to review this well-written paper

Minor comments needs to be dealt with

1. The author has reference 21 which was not cited anywhere in the article
2. Line 475, the author used Vancouver referencing throughout the document, however, on this line they did not do so.
3. Line 486 beginning, there’s a typo error

Well done to the authors!!!
