# Supplementary material for: Retrospective in silico mutation profiling of SARS-CoV-2 structural proteins circulating in Uganda by July 2021: Towards refinement of COVID-19 disease vaccines, diagnostics, and therapeutics
Source: PLoS One. 2022 Dec 22;17(12):e0279428. doi: 10.1371/journal.pone.0279428 (PMC9778641; doi:10.1371/journal.pone.0279428)
Supplement: Attachment — Submitted filename: Response to Reviewers.doc [file pone.0279428.s008.doc]

**Fax: +256-414-554685**

**E-mail: bds@covab.mak.ac.ug**

**MAKERERE UNIVERSITY**

**COLLEGE OF VETERINARY MEDICINE, ANIMAL RESOURCES & BIOSECURITY**

**P.O. Box 7062 Kampala, Uganda**

**Tel: +256-414-554685**

**SCHOOL OF BIOSECURITY, BIOTECHNICAL AND LABORATORY SCIENCES**

**DEPARTMENT OF BIOTECHNICAL & DIAGNOSTIC SCIENCES**

Your Ref:	Our Ref:	Date: 4^th^ December 2022

**Response to the Academic Editor and the Reviewers:**

| **SN** | **Item:** | **Comment** | **Response** |
| --- | --- | --- | --- |
| A. | Editor’s comments | 1. Please ensure that your manuscript meets PLOS ONE's style requirements, including those for file naming. | We have ensured strict adherence to all the PLOS ONE’s style requirements throughout the manuscript including file naming |
|  |  | 2. Please provide additional details regarding participant consent. In the ethics statement in the Methods and online submission information, please ensure that you have specified what type you obtained (for instance, written or verbal, and if verbal, how it was documented and witnessed). If your study included minors, state whether you obtained consent from parents or guardians. If the need for consent was waived by the ethics committee, please include this information. | This was a “nested study” under “master study” titled “*Development and validation of multiplex lateral flow assay for detection and differentiation of from SARS-CoV-2 and other human coronaviruses*”, **ref. no. SBS-2021-38**. Because the entire study used secondary data (including the sequence dataset analyzed), we were granted consent waiver by the Makerere University College of Health Sciences (MAKCHS) School of Biomedical Sciences Research Ethics Committee. Please find accompany consent waiver attached.  We have now modified the Ethics statement by incorporating additional statement on consent waiver. This statement has been included in the Method section of the **Manuscript without track changes** (line 211-216.), and it has been submitted online. |
|  |  | 3. Please review your reference list to ensure that it is complete and correct. If you have cited papers that have been retracted, please include the rationale for doing so in the manuscript text, or remove these references and replace them with relevant current references. Any changes to the reference list should be mentioned in the rebuttal letter that accompanies your revised manuscript. If you need to cite a retracted article, indicate the article’s retracted status in the References list and also include a citation and full reference for the retraction notice. | We have thoroughly revised citations in the text against bibliography, therefore as it stands, there are no mismatches. |
|  |  | 4. Include the entropy value in the y axis for Figure 2A | Initially, we used graphs generated automatically by the *BioEdit*. However, BioEdit does not offer interactive interface, which does not allow manual manipulation by the end users. To include entropy values in the y-axis of Figure 2A, re-plotted all the graphs in Figure 2A-D graphs using the *Microsoft Excel version 2019*. |
|  |  | 5. Please list the sources and version of all software used in the methodology eg Muscle | Initially, the sources and/or versions of some software used in the study were not mentioned. We have rectified this omission by ensuring that all the software mentioned have sources and version numbers. |
|  |  | 6. The quality of the figures are poor | We strived to ensure that the current version of the figures will meet the taste of our readers. The quality of the figures was improved by increasing the dpi from the 96 to 600. |
|  |  | 7. Revise phylogenetic tree (Figure 5): Exclude bootstrap value less than 70% and collapse some of the branches to improve the figure | We acknowledge your concern on improving the clarity of the figure by collapsing some of the branches. However, collapsing the branches of the tree to some extent distorted the formation contained therein given that each of the OTUs is a separate entity representing 141 spike sequence variants encountered. Therefore, to avoid losing information, we decided to leave the figure intact; however, we excluded it for the body of the manuscript and instead included it as supporting information (S2 Fig.). |
|  |  | 8. The abstract need to be rewritten to also include the method used. The abstract is not attractive; it should arouse curiosity in the reader and should be clearer. It should not start with ‘Whereas” | We highly appreciate this suggestion. We re-wrote the abstract to address this concern. |
|  |  |  |  |
| B. | Reviewer # 1 comments | 1. Reference number 21 is omitted in the content. | This reference was omitted in error when we were rearranging the bibliography. Instead of progressing from Bibliography listed 20 to 21, we erroneously repeated 20. This omission has now been addressed in the manuscript **line 123**. |
|  |  | 2. Line 475 reference using the number like in content. | This was an error, *Bugembe et al 2021* is listed **64** in the manuscript’s Bibliography. This omission has now been corrected in the manuscript **line 489**. |
|  |  | 3. Line 486 spelling of beginning should be corrected. | The misspelling of the word “beginning” was a typographical error. We have corrected it as indicated in the manuscript **line 500**. |
|  |  |  |  |
| C. | Reviewer # 2 comments | 1. The author has reference 21 which was not cited anywhere in the article. | This omission has been revised as explained in the above (Reviewer #1, comment 1) |
|  |  | 2. Line 475, the author used Vancouver referencing throughout the document, however, on this line they did not do so. | This omission has been revised as explained in the above (Reviewer #1, comment 2) |
|  |  | 3. Line 486 beginning, there’s a typo error | This spelling error has been revised as explained in the above (Reviewer #1, comment 3) |

Steven Odongo (Ph. D)

[**Tel:** +82](tel:+82)326264332

**Mob:** +821062701977/+256778660020

**E-mail:** [steven.odongo@mak.ac.ug](mailto:steven.odongo@mak.ac.ug) / [opodongo@yahoo.co.uk](mailto:opodongo@yahoo.co.uk) / [steven.odongo@ghent.ac.kr](mailto:steven.odongo@ghent.ac.kr)
